# My First Christmas in Hospital

**Published:** 1914-01-03

**Authors:** 


					oi/5- t /K.
VVx^Cfc/J
jyv>?, X ui-.
OsQ-'3 cyta&Aj <j\ksy\^J La? /vwjs_,
3 %JL, clasps Ih^c-UL 2-Cryyix. V6^X)uaX^u^
^$io?l^ OsvJL mcltiK. cwtw?c( v/i- -
C3 avj, ?\ <v>*_cxo womwvo/ uHslk
cM> \j-za*?<&ajJjlcL crot/i o-iASi* &Oxsfa ?.
-VAM^O Q/yvoi' ~tAc -fi/tfcCi. ^IjvLiO utMa-cA,
fvoxl IntM- ixMxisU O-USL, ^k&O
\/fU XivjL rrU^J^- <-& ou^H/,
W-K/Le OWI J^TXO-
s-miaTv <zJ Azrtl. Hhi'- c^asyyi&jduyvvL. .
( u.-^vvciv fuxds c*yvav>cc{ t,iuL- .ywgAfc
'^e^-criX' ) <*jxlo fwA, a-vu tcr? .
i t> t-lvULC cr cAxreM ia^
~ eAorcOv q)*Qubi/xfsls /yyUX/* A/VVcC O^^?
Kio aXu/vusLc^nXo, un- ^"xAx, o/uxAj?-
^xOU'l^XC^/'-^ |ww- Cl/C2v t/VV Ivo-ru)tt>w*
cCa-vUJlL cct/vrui a^nai oJX^X. ^zsastw
c t'nAo "0^ oJ^i. c| w>? < J oJXEPriydjasvUo
,c/? , ci fut- l/?? as^rjL^
J ???*. vRi-<^- 3^uyyy~, (W-xsi -?u. JVlcui
JtoJttly.. cl ~n?/vv tluyic Vv-txo Cjja, 0xvv-o\.
C^V-a.eiWoO - {3^vxi_ cv-j^ OULcIyi/)
wrCt/5 yrjLJXn^K.^ Ua>*0- 51.1 Jv. ~Co<V-X-<^
jl zs^j?^ vu^s^03
?C7^o|Jtjyt'. C5 Kt/W t*J~C ia>-c/V\0
Vy M/ci~ cx^Ca/v Ox ir?/^ c^a/^'
SckJih &>4xua,
cA CjX' 10 yj)JlSL/} .
Great Ormond Street Hospital.
[The above needs no introduction, and that none of rts
freshness or genuine originality might be lost we have
reproduced the actual letter of the young patient at Great
Ormond Street, which was sent to us with the adult viow
of the same proceedings which appears on the next page,?>
Ed.] ? , -!. 1 ? '???':u-: ???

				

